# Biohybrid Bovine Bone Matrix for Controlled Release of Mesenchymal Stem/Stromal Cell Lyosecretome: A Device for Bone Regeneration

**DOI:** 10.3390/ijms22084064

**Published:** 2021-04-14

**Authors:** Elia Bari, Ilaria Roato, Giuseppe Perale, Filippo Rossi, Tullio Genova, Federico Mussano, Riccardo Ferracini, Marzio Sorlini, Maria Luisa Torre, Sara Perteghella

**Affiliations:** 1 Department of Drug Sciences, University of Pavia, Viale Taramelli 12, I-27100 Pavia, Italy; elia.bari@unipv.it (E.B.); sara.perteghella@unipv.it (S.P.); 2 Department of Surgical Sciences, CIR-Dental School, University of Torino, Via Nizza 230, I-10126 Torino, Italy; ilaria.roato@unito.it (I.R.); federico.mussano@unito.it (F.M.); 3 Industrie Biomediche Insubri SA, Via Cantonale 67, CH-6805 Mezzovico-Vira, Switzerland; giuseppe@ibi-sa.com; 4 Faculty of Biomedical Sciences, University of Southern Switzerland (USI), Via G. Buffi 13, CH-6900 Lugano, Switzerland; 5 Ludwig Boltzmann Institute for Experimental and Clinical Traumatology, Donaueschingenstrasse 13, A-1200 Vienna, Austria; 6 Department of Chemistry, Materials and Chemical Engineering “Giulio Natta”, Politecnico di Milano, Via Mancinelli 7, I-20131 Milano, Italy; filippo.rossi@polimi.it; 7 Department of Life Sciences and Systems Biology, University of Torino, Via Accademia Albertina 13, I-10123 Torino, Italy; tullio.genova@unito.it; 8 Department of Surgical Sciences and Integrated Diagnostics, University of Genova, Viale Benedetto XV 6, I-16132 Genova, Italy; ferracini@edu.unige.it; 9 SUPSI—Department of Innovative Technologies, Lugano University Centre, Campus Est, Via la Santa 1, CH-6962 Viganello, Switzerland; marzio.sorlini@supsi.ch; 10 PharmaExceed Srl, Piazza Castello 19, I-27100 Pavia, Italy

**Keywords:** bone grafting, mesenchymal stem cells, MSC-secretome, MSC-extracellular vesicles, MSC-exosomes, bone regeneration

## Abstract

SmartBone^®^ (SB) is a biohybrid bone substitute advantageously proposed as a class III medical device for bone regeneration in reconstructive surgeries (oral, maxillofacial, orthopedic, and oncology). In the present study, a new strategy to improve SB osteoinductivity was developed. SB scaffolds were loaded with lyosecretome, a freeze-dried formulation of mesenchymal stem cell (MSC)-secretome, containing proteins and extracellular vesicles (EVs). Lyosecretome-loaded SB scaffolds (SBlyo) were prepared using an absorption method. A burst release of proteins and EVs (38% and 50% after 30 min, respectively) was observed, and then proteins were released more slowly with respect to EVs, most likely because they more strongly adsorbed onto the SB surface. In vitro tests were conducted using adipose tissue-derived stromal vascular fraction (SVF) plated on SB or SBlyo. After 14 days, significant cell proliferation improvement was observed on SBlyo with respect to SB, where cells filled the cavities between the native trabeculae. On SB, on the other hand, the process was still present, but tissue formation was less organized at 60 days. On both scaffolds, cells differentiated into osteoblasts and were able to mineralize after 60 days. Nonetheless, SBlyo showed a higher expression of osteoblast markers and a higher quantity of newly formed trabeculae than SB alone. The quantification analysis of the newly formed mineralized tissue and the immunohistochemical studies demonstrated that SBlyo induces bone formation more effectively. This osteoinductive effect is likely due to the osteogenic factors present in the lyosecretome, such as fibronectin, alpha-2-macroglobulin, apolipoprotein A, and TGF-β.

## 1. Introduction

Bone tissue, the skeleton’s main constituent, is a highly specialized mineralized connective tissue with important structural and metabolic functions. Through a process called “bone remodeling”, bone possesses the intrinsic capacity for regeneration as part of the repair response to injury, skeletal development, and continuous remodeling throughout adult life [1].

Sometimes, bone regeneration’s physiological process is impaired or insufficient due to infections, osteonecrosis, trauma, tumors, or inherent genetic disorders, leading to bone loss [2]. In these cases, bone grafting is a therapeutic strategy used to fill the bone gap, provide support, and enhance the defect’s biological repair, stimulating new bone formation at the receiving site. Currently, bone grafting is commonly performed in various orthopedic, oral/dental, and maxillofacial procedures; it is estimated that over two million bone-grafting procedures are performed worldwide every year [3]. Autologous bone grafting involves the transplant of fresh cortical or trabecular bone (or a combination of both) from one site in the body to another within the same patient. It is still considered the “gold standard” for bone replacement due to its low risk of immunological rejection and high histocompatibility, as well as osteoconductive and osteoinductive properties [4]. However, autograft has several limitations, including donor-site morbidity and availability, which may be partly overcome by using an allogeneic bone graft. In this case, cortical/trabecular bone is transplanted from cadavers into the patient. Allogenic bone graft maintains osteoconductivity and has limited osteoinductivity, but it may be a vector for disease transmission or bacterial infection [5]. Therefore, new bone reconstruction solutions are needed, and several bone substitutes of biological and synthetic origins are available [6]. Synthetic materials, such as hydroxyapatite (HA), bioactive glasses, and β-tricalcium phosphate (TCP), are primarily proposed as they are similar to the bone mineral matrix [7], even if they do not have the same biological and mechanical properties compared with bone. In this regard, 3D printing applied to such materials has recently been proposed as an innovative technique for personalized bone substitute therapy, promoting translation to clinical practice [8,9]. On the other hand, animal-derived materials (xenografts) may provide structures similar to living tissues and induce specific cellular responses. Interestingly, it is also possible to combine natural and synthetic components, yielding more favorable outcomes and tuning mechanical and physical properties. In this regard, among modified xenografts, SmartBone^®^ is a biohybrid bone substitute composed of a bovine bone-derived matrix mechanically reinforced with poly(l-lactide-*co*-ε-caprolactone) (PLCL) and integrated with RGD-containing collagen fragments to improve the cell viability, the hydrophilicity of the matrix, and the overall biocompatibility [3,10]. It has to be underlined that such a scaffold is the result of the development of a technological platform that can serve as bone regeneration under various applications, including drug and molecule delivery. It is noteworthy that SmartBone^®^ is also available as a new CE-labeled class III medical device, and it has a vast clinical record being also commercially available as a bone substitute for bone regenerative surgeries in all skeletal districts for oral, maxillofacial, orthopedic, and oncologic indications [11,12,13,14]. This allows the current developments to have a robust path into translating research from bench to bed and to market finally [15].

There is growing interest in combining bone xenografts with cellular components, e.g., mesenchymal stem cells (MSCs), to promote osteoinductivity and osteoinductivity and significantly improve bone repair and regeneration [16]. MSCs are multipotent stromal stem cells that can be harvested from many different sources, e.g., adipose tissue and bone marrow, and that possess regenerative ability, as well as immunomodulatory capacity. Previous studies have reported that MSCs, combined with bone substitutes, can regenerate bone defects [17,18,19,20], and their activity has been attributed to regenerative properties linked with cell differentiation. However, more recently, MSCs have been discovered to act as “cell factories” that secrete various bioactive molecules and extracellular vesicles (EVs), which work on neighboring cells and tissues by exerting paracrine effects [21,22]. With the use of the MSC-secretome in tissue regeneration, the problems linked to MSC-based therapies, such as low cell survival and engraftment, would be circumvented. Moreover, it would bring technological advantages, such as the availability of an off-the-shelf product and the ease of storage/transport/use in outpatient settings. In this regard, the group led by Prof. Torre recently moved forward with the clinical application of MSC-secretome, proposing a standardized and ready-to-use freeze-dried injectable powder named lyosecretome. The lyosecretome production process is compliant with Good Manufacturing Practice (GMP) [23,24,25,26].

In this context, the present study proposes the enrichment of SmartBone^®^ grafts with lyosecretome, intended for the in vivo controlled release of MSC paracrine factors in bone regenerative medicine. The lyosecretome formulation was optimized to achieve proper and homogeneous loading of proteins and EVs into SmartBone^®^ scaffolds via an adsorption method. After loading, the scaffolds were characterized in terms of morphology, drug loading, and drug release. Lastly, the ability of secretome-enriched SmartBone^®^ (SBlyo) to induce adipose tissue-derived stromal vascular fraction (SVF) differentiation and new healthy bone tissue formation was investigated.

## 2. Results and Discussion

In previous studies, the regenerative potential of adipose-derived MSCs and SVF on SmartBone^®^ (Industrie Biomediche Insubri SA, Mezzovico-Vira, Switzerland) grafts were previously reported [27]. In this work, we investigated the ability of MSC-lyosecretome to promote osteogenic differentiation of SVF on the same bone scaffold. The combination of MSC-lyosecretome with tissue engineering strategies may result in next-generation osteoinductive scaffolds that can improve bone formation speed and quality. MSC-secretome was prepared according to GMP-compliant procedures, and it was formulated into a standardized and ready-of-the-shelf product, named lyosecretome [23,24,25,26]. Lyosecretome contained 30.72 ± 2.139 µg of proteins and 1.62 ± 0.0329 µg of lipids per mg of powder. The EV mean diameter was 170.9 ± 9.2 nm, and the d_10_, d_50_, and d_90_ were 100.4 ± 2.8, 142.1 ± 7.2, and 308.5 ± 32.3 nm, respectively.

**Figure 1:**
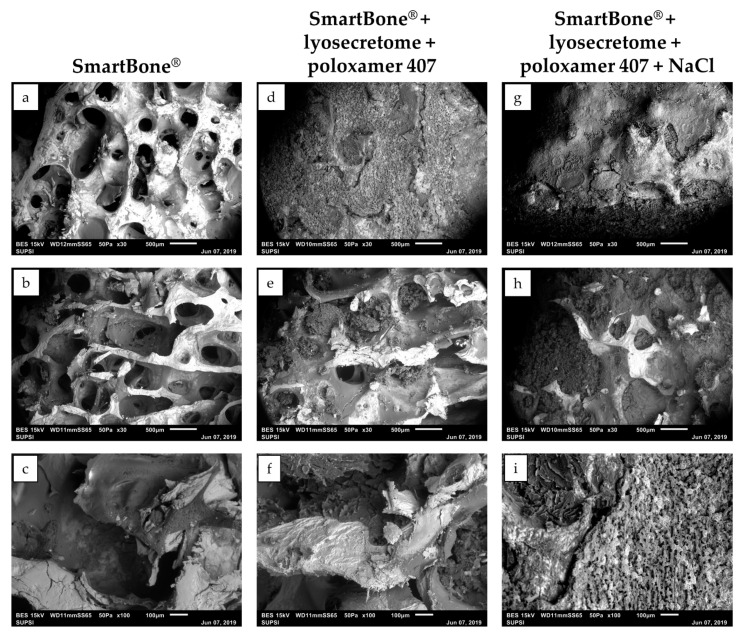
Morphological investigation by ESEM of SmartBone^®^ (**a**–**c**) and SmartBone^®^ + lyosecretome scaffolds with (**d**–**f**) and without (**g**–**i**) NaCl before drug release studies. SEM images were taken at increasing magnifications (30× and 100×). Scale bar: 500 and 100 µm.

**Figure 2:**
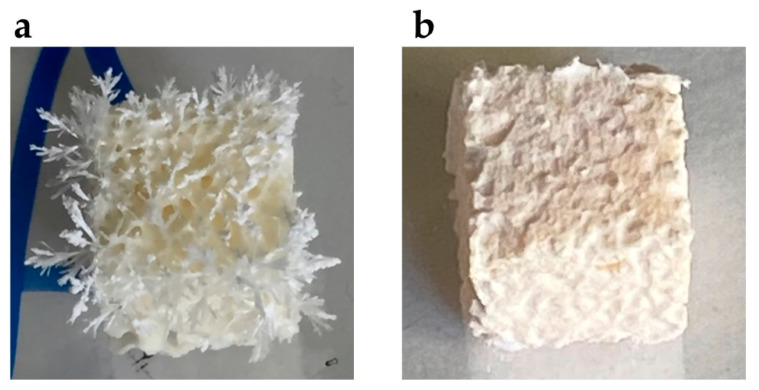
Crystallization of mannitol during the freeze-drying step (**a**) was prevented by the addition of NaCl (**b**).

Lyosecretome was loaded onto SmartBone^®^ scaffolds via an adsorption method since it is straightforward and cost-effective [28]. Scaffolds were immersed in a lyosecretome solution and then freeze-dried. For effective and homogenous loading, the lyosecretome formulation needed to be optimized. Initially, Lutrol^®^ F127 (poloxamer 407) was added to the lyosecretome solution to increase the wettability of SmartBone^®^ and, thus, increase the liquid penetration into the inner core, functional to achieve a homogeneous loading of secretome proteins and lipids onto all free surfaces of the grafts. Noteworthy, poloxamer 407 was also shown to prevent/reduce the propensity for peptides unfolding, thus promoting, with mannitol, the stabilization of secretome proteins [29,30]. ESEM’s morphological characterization revealed the deposition of the “active ingredient” coating over the material’s surfaces (Figure 1d–f). The external pores were almost completely closed, as well as the interior ones (with some exceptions in the center, Figure 1e). The visible material deposition was due to lyosecretome, as the only poloxamer-loaded SB showed no material deposition (see Figure S1 in the Supplementary Materials). However, the formulation needed to be further optimized, as the lyosecretome coating on SmartBone^®^ appeared fragile and brittle; moreover, during the freeze-drying step, needle-like crystals emerged from the surface of the scaffold (Figure 2) because of mannitol crystallization. To prevent mannitol crystallization, NaCl was added as an excipient [31]. Using a formulation with poloxamer 407 and NaCl, uniform deposition of lyosecretome was observed on the surface of the support structure (Figure 1g), with a complete closure of the external and the central pores (Figure 1h,i). Therefore, the combination of poloxamer and NaCl (SBlyo) was selected as most appropriate to manufacture samples for in vitro testing.

**Figure 3:**
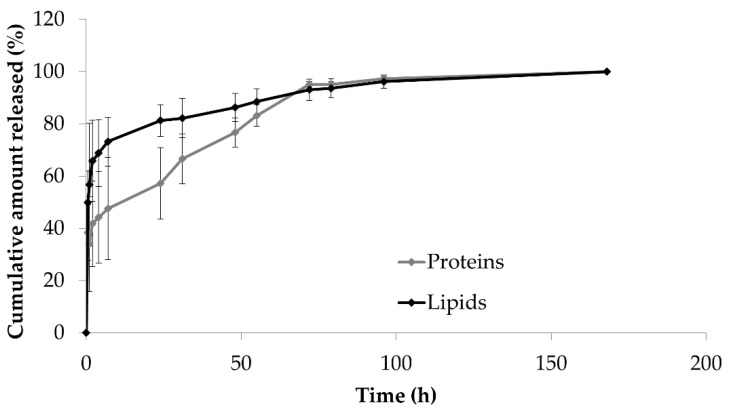
In vitro protein and lipid release profiles from SBlyo scaffolds immersed in pH 7.2 PBS at room temperature. Mean values ± standard deviation, *n* = 3.

The average loading of SBlyo scaffolds was 1230.8 ± 283.677 µg for proteins and 81.38 ± 30.44 µg for lipids (mean value ± standard deviation, *n* = 3). The different porosity among the different scaffolds likely led to the absorption of different amounts of lyosecretome. In light of future scale-up and translatability toward the clinical stage, this method’s loading process should be further improved to reduce the high batch-to-batch variability. Protein and lipid release profiles are shown in Figure 3. The cumulative release from SBlyo scaffolds showed an initial burst release, within 30 min, of 39% for proteins and 50% for lipids. Then, proteins were gradually released from SB, reaching 95% after 72 h. A faster release was observed for lipids; after 24 h, 81% of lipids were released (vs. the 57% of proteins). At the end of the release studies, the ESEM investigation revealed the secretome’s complete release from the scaffold (data not shown). The burst effect, typically Fickian, can be explained considering that the formulation components (mannitol, poloxamer 407, and NaCl) easily recall the PBS into the scaffold that immediately dissolves the external layers of lyosecretome deposited on SB. Proteins are then slowly released compared to lipids as they are probably more strongly adsorbed on the surface of SBs. It is worth noting that this release behavior was flipped (proteins vs. lipids) with respect to polycaprolactone and alginate 3D printed scaffolds loaded with lyosecretome [9]. This may result from the high affinity of proteins for the bone-derived matrix and the RGD-containing collagen fragments of SB scaffolds.

The drug release data were further processed by elaborating the release’s kinetic model; the employed models were Higuchi, Peppas–Sahlin, Ritger–Peppas, and zero-order. Table 1 lists the in vitro release model results fitting for both proteins and lipids from SBlyo. Low *R*^2^ values were observed for each model considered, indicating that the release of proteins and lipids from SBlyo is not mainly governed by diffusion or erosion.

**Figure 4:**
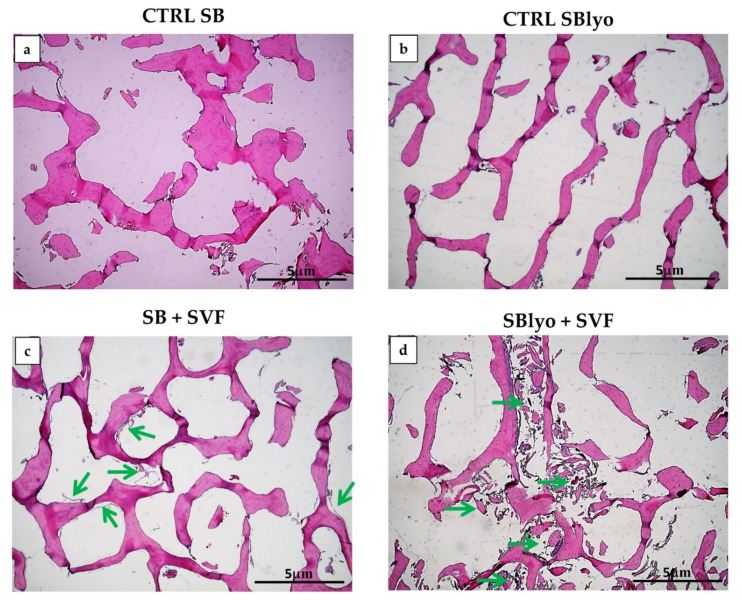
Growth of cells on SB and SBlyo after 14 days. H&E staining shows empty trabeculae of SB (**a**) and SBlyo (**b**) without cell seeding (CRTL), whereas the initial growth of SVF cells on SB (**c**) and SBlyo (**d**) was evident (green arrows). Magnification 4×. Scale bar: 5 μm.

The tissue growth of SVF plated on SB and SBlyo was evaluated at 14 and 60 days. H&E staining was performed, showing the trabecular structure of SB and SBlyo without cells (Figure 4a,b, respectively) and the SB colonization by SVF, showing an initial new tissue formation at 14 days (Figure 4c,d), mainly on SBlyo.

**Figure 5:**
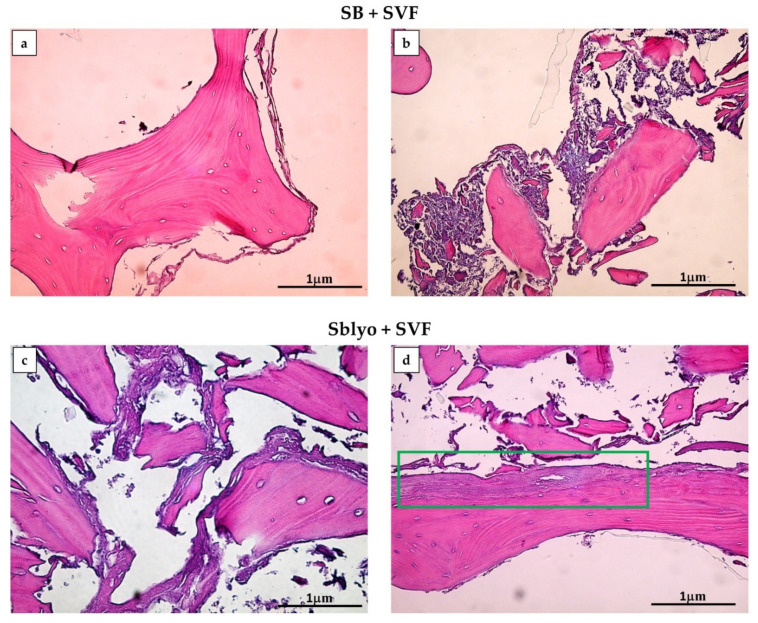
H&E staining. New tissue formation is evident on both SB (**a**,**b**) and SBlyo (**c**,**d**) after 60 days of culture. Panels (**a**,**b**) show cell growth in the periphery and among bone trabeculae, respectively. On SBlyo (**d**), a well-organized tissue portion is visible. The green rectangle indicates an area with well-organized neo-formed tissue. Magnification 20×. Scale bar: 1 µm.

After 60 days, new bone tissue was evident in both SB and SBlyo (Figure 5a,d). In all samples, SVF colonized and formed new tissue, starting from the periphery (Figure 5a) of the SB and progressively filling bone lacunae (Figure 5b). These observations are in accordance with previously reported results [3,27] and confirm the ability of MSCs to colonize and grow on SB, as also evidenced in clinical studies [10,11,33]. However, even though the neo-tissue formation area was detectable on both SBs, it was more marked on SBlyo than SB, as shown in Figure 4c,d, respectively. Since all SBs were plated with the same SVF and cultured in OM, the major formation of well-organized bone tissue is attributable to the presence of lyosecretome and, thus, to the paracrine factors and EVs. Accordingly, other researchers reported that MSC-EVs added to scaffold promoted robust bone formation [34,35,36].

**Figure 6:**
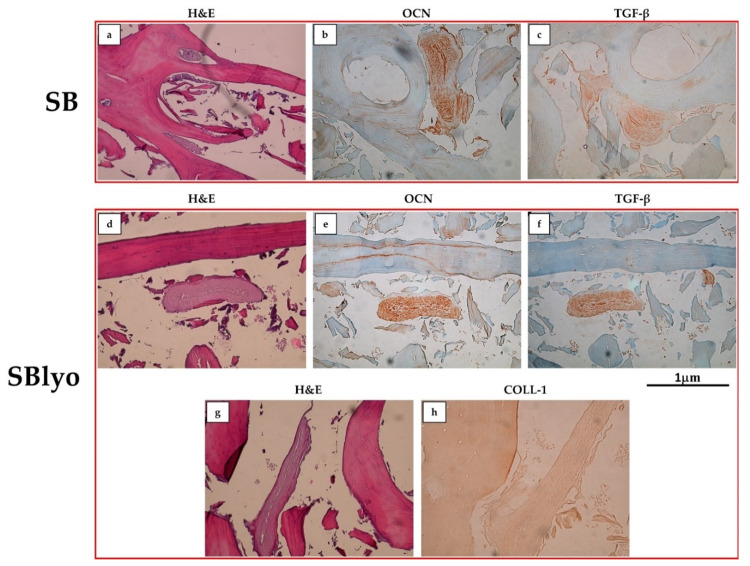
H&E, OCN, and TGF-β staining performed on SB (**a**–**c**) and SBlyo (**d**–**h**) at 60 days. Magnification 20×. Scale bar: 1 µm.

In both SB and SBlyo, tissue formation was observed through H&E staining, along the trabeculae’s entire perimeter and within the lacunae (Figure 6a,d,g), indicating that the SmartBone^®^ scaffold can stimulate the proliferation and differentiation of MSCs into osteoblasts. To further confirm this result, the expression of COLL-1, OCN, and TGF-β on both SB and SBlyo was assessed at 60 days. On SB, OCN and TGF-β resulted highly expressed (Figure 6b,c), whereas COLL-1 was weakly positive (data not shown). On SBlyo, a large area of tissue in which OCN and TGF-β are highly expressed was present (Figure 6e and Figure 6f, respectively), while COLL-1 markedly stained the new tissue on SBlyo (Figure 6h).

**Figure 7:**
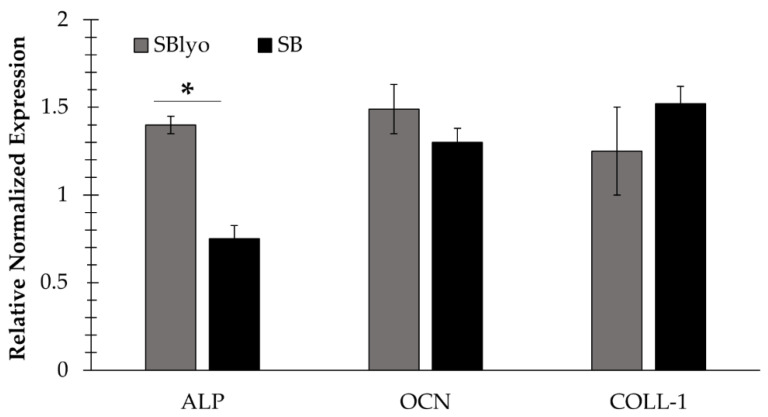
Expression of mature osteoblast markers (ALP, COLL-1, and OCN): marker expression of cells grown on SBlyo (gray) and those grown on untreated SB (black). “*” indicates a significant difference between SB and SBlyo (*p* < 0.05).

To quantify the expression of genes typically expressed by osteoblasts, such as ALP, OCN, and COLL-1, RNA was extracted from cells grown on SB and SBlyo after 30 and 60 days (Figure 7). A low yield of RNA was obtained, even though different techniques of RNA extraction were tested. The ALP expression was significantly higher in cells on SBlyo than SB; instead, a nonsignificant difference in the COLL-1 and OCN marker expression was observed, even though the trend of expression was higher in SBlyo than in SB. These data confirm the previously reported potential of SVF to differentiate into osteoblasts when cultured in OM on SB [27] and suggest the ability of lyosecretome to further promoting osteodifferentiation.

**Figure 8:**
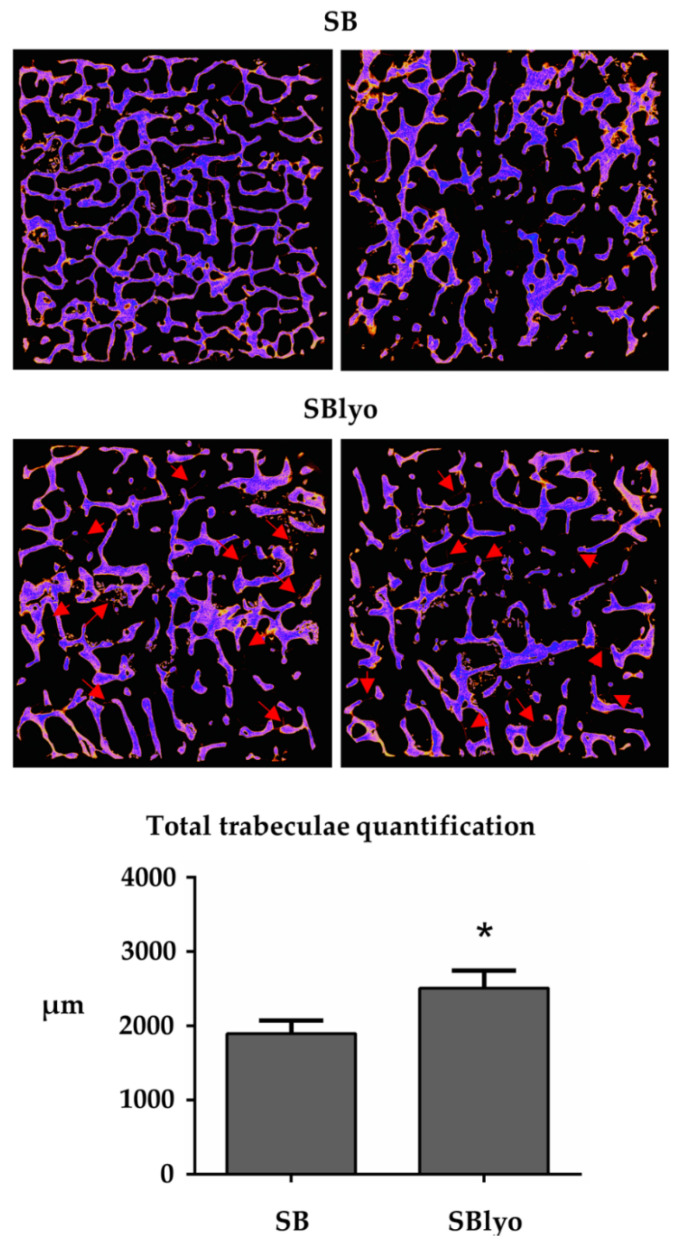
Trabeculae quantification. The neo-trabeculae are visible as red filaments (indicated by the red arrows). “*” indicates a significant difference between SB and SBlyo (*p* < 0.05).

To evaluate the new tissue mineralization rate formed on SB and SBlyo, we analyzed the scaffold through micro-CT analysis, which quantifies the newly formed trabeculae. Since the mineralization process takes a long time, quantification of newly formed trabeculae is only possible after 60 days. Figure 8 shows the thickness of the new mineralized trabeculae formed by the cells inside the SB and SBlyos scaffolds. By comparing the two values, it is possible to observe a significant increase of newly formed trabeculae inside SBlyo of about 20% compared to SB (*p* < 0.05).

Overall, such results confirm the role of MSC-secretome as an intercellular messenger able to orchestrate bone regeneration. Accordingly, previous studies showed that, in rat models of bone defects, treatment with MSC-secretome results in bone regeneration [37,38,39,40], as well as increases matrix mineralization [41,42] and vascularization [25,29]. This intriguing osteodifferentiating effect of the lyosecretome likely depends on its osteogenic protein composition. The proteomic characterization of lyosecretome has been reported in previous work [23]. Among the most represented proteins in the lyosecretome, fibronectin, alpha-2-macroglobulin, apolipoprotein A, and TGF-β have pivotal roles in the osteogenesis process. It has been suggested that fibronectin participates in bone formation. In detail, Moursi and colleagues demonstrated that fibronectin regulates both normal morphogenesis and gene expression of osteoblasts during the early stages of differentiation. Likely, by blocking fibronectin, mRNA expression for alkaline phosphatase and osteocalcin genes was suppressed [43]. Accordingly, Yunyi and colleagues demonstrated that fibronectin induced the early onset of osteogenic differentiation in murine embryonic stem cells and resulted in threefold higher calcium deposition at day 11 of osteogenic culture compared to the control group [44]. Alpha-2-macroglobulin was shown to enhance the immediate availability of the osteogenic growth peptide (OGP), which is complexed noncovalently to heat-sensitive, high-molecular-weight OGP-binding proteins (OGPBPs) that in turn stimulate bone formation [45]. Recent evidence suggested a link between bone mass and high-density lipoprotein (HDL), where imbalances in lipid metabolism affect bone mass and quality. In this regard, it has been observed in mice that deficiency of apolipoprotein A generates changes in the bone cell precursor population, decreasing osteoblast production and resulting in reduced bone mass and impaired bone quality [46]. Lastly, TGF-β plays a critical role in bone remodeling, stimulating matrix protein synthesis, and regulating the cells responsible for bone formation and resorption. Indeed, as recently reviewed [47], TGF-β can induce differentiation and proliferation of osteoblasts, the cells responsible for the construction of new bone; on the other hand, it can inhibit the formation of osteoclast precursors and bone resorption and can have inhibitory effects on osteoclasts, the cells responsible for bone resorption.

Future studies can investigate bone fracture healing under the application of SBlyo under different bone fracture geometries and loading conditions. This can be facilitated using computational modeling and incorporation of advection/diffusion, cell proliferation, and differentiation into the model, as reported in [48,49].

## 3. Materials and Methods

### 3.1. Materials

Cell culture media and antibiotics were purchased from Euroclone (Milan, Italy). A commercial platelet lysate kit (PL) was obtained from Carlo Erba Reagents (Milan, Italy). Collagenase NB4 was purchased from SERVA Electrophoresis, Heidelberg, Germany. MicroDec EDTA-based was purchased from Diapath, Martinengo, Italy. The EZ Prep Concentrate solution and the Universal DAB Detection Kit were purchased from Ventana Medical Systems Inc., Tucson, AZ, USA. Mouse monoclonal antibodies were obtained from Abcam (Milan, Italy). Acetone, beta-glycerophosphate, bovine serum albumin (BSA), formaldehyde, Kaiser’s glycerol gelatin, Mayer’s hematoxylin solution, mannitol, phosphate-buffered saline (PBS), phosphatidylcholine (PC), poloxamer 407 (Lutrol^®^ F127), and sodium chloride were purchased by Sigma Aldrich (Milan, Italy). All reagents were of analytical grade. SmartBone^®^ scaffolds (1 cm × 1 cm × 0.3 cm in size) were provided by Industrie Biomediche Insubri SA (Mezzovico-Vira, Switzerland).

### 3.2. Lyosecretome Preparation and Characterization

#### 3.2.1. Lyosecretome Preparation

Freeze-dried MSC-secretome (lyosecretome) was prepared and characterized according to previously reported procedures compliant with current Good Manufacturing Practice (GMP) [23,24,25,26]. Briefly, adipose tissues were collected from patients undergoing abdominoplasty surgery after informed consent (ASST Grande Ospedale Metropolitano Niguarda, Milan, Ref. 12 November 2009). Adipose-derived MSCs (AD-MSCs) were harvested from adipose tissues [50,51], seeded into flasks (10,000 cells/cm^2^) at 37 °C and 5% CO_2_, and expanded in DMEM/F12 minimal medium plus 5% *v*/*v* PL, plus 1% *v*/*v* penicillin/streptomycin and 1% *v*/*v* amphotericin B until P3. The release of secretome was induced by culturing MSCs in DMEM/F12 without platelet lysate (lysate starvation) for 48 h. MSCs used fulfilled the requirements needed for clinical use in terms of identity (as stated by the International Society for Cellular Therapy [52]) and sterility (according to Eu. Ph. 9.0, 2.6.27).

Cell culture supernatants were centrifuged at 3500× *g* for 10 min and then ultrafiltered by tangential flow filtration (KrosFlo^®^ Research 2i system, Spectrum Laboratories, Milan, Italy) using a filtration module with a superficial area of 235 cm^2^ and a molecular weight cutoff (MWCO) of 5 kDa (Spectrum Laboratories, Milan, Italy). First, samples were concentrated at 0.5 × 10^6^ cell equivalents per mL (calculated as total cell number/mL of supernatant) and then diafiltered using sterilized ultrapure water as a buffer. Once concentrated and purified, mannitol (0.5% *w*/*v*) was added to the secretome, which was then frozen at −80 °C and freeze-dried (Christ Epsilon 2–16D LSCplus) at 8 × 10^−1^ mbar and −50 °C for 72 h. The obtained lyosecretome was stored at −20 °C until use (12 months). Each mg of lyosecretome corresponds to 0.1 × 10^6^ cell equivalents (calculated as total cell number/milligrams of lyosecretome).

#### 3.2.2. Lyosecretome Characterization

##### Total Proteins and Lipids

A BCA Protein Assay Kit (Thermo Fisher Scientific, Milan, Italy) was used to quantify proteins in the lyosecretome. The working reagent solution was prepared as specified by the manufacturer, added to each sample (or standard) in a 1:1 ratio, and incubated at 37 °C for 2 h. The absorbance was measured at 562 nm with a microplate reader (Synergy HT, BioTek, Swindon, UK). A calibration curve with *R*^2^ = 0.99 was prepared using standard protein solutions (BSA).

Total lipids were dosed by Nile Red [23]. Nile Red was solubilized in acetone (3.14 M), diluted 100× in PBS, and incubated with samples (1:9 ratio) for 5 min at room temperature. The fluorescence was measured by Synergy HT at fixed wavelengths (530/25 excitation and 645/40 emission). A calibration curve with *R*^2^ = 0.99 was prepared using standard lipid solutions (PC). All analyses were conducted in triplicate.

##### EV Particle Size and Concentration

The particle size and concentration of EVs were determined by nanoparticle tracking analysis (NTA, NanoSight NS 300 equipment, Malvern Instruments, Malvern, UK). First, 1 mg of lyosecretome was dispersed in 1 mL of deionized water and analyzed. Measurements were carried out at room temperature with a detection angle of 90°, and data were elaborated using the NTA software. All analyses were conducted in triplicate.

### 3.3. Lyosecretome-Loaded SmartBone^®^ (SBlyo) Preparation and Characterization

#### 3.3.1. SBlyo Preparation

Each SmartBone^®^ sample (1 cm × 1 cm × 0.3 cm in size) was loaded with 16,000 cell equivalents of lyosecretome. SmartBone^®^ samples were placed in a 24 Multiwell and covered with an aqueous solution containing lyosecretome (1.7 mg/mL), poloxamer 407 (Lutrol^®^ F127), and NaCl (0.1% *w*/*v* each) for 1 h at 4 °C. Samples were then frozen at −80 °C and freeze-dried (Christ Epsilon 2–16D LSCplus) at 8 × 10^−1^ mbar and −50 °C for 24 h. All the procedures were performed by qualified operators equipped with protective and disposable clothes in aseptic conditions in a grade B cleanroom suite to avoid bacterial contamination.

#### 3.3.2. Drug Loading

The amount of secretome loaded was determined by dispersing SmartBone^®^ samples in deionized water under magnetic stirring for 168 h. Protein and lipids were dosed in the final solution, as reported in Section 3.2.2. Each experiment was performed in triplicate.

#### 3.3.3. Morphological Investigation by Environmental Scanning Electron Microscopy (ESEM)

Samples for ESEM analysis were previously sectioned with a steel blade to observe their inner structure. ESEM analysis was performed at 10 kV with 50 EP Instrumentation (Zeiss, Jena, Germany), according to previously published methodology [33].

#### 3.3.4. Drug Release Studies

SBlyo scaffolds were immersed in 5 mL of pH 7.2 phosphate-buffered saline (PBS, USP) at room temperature. At fixed time intervals, 500 µL of the release medium was collected and replaced by an equivalent amount of fresh PBS. The lipid and proteins in the release media were dosed by Nile Red and BCA assay, respectively, as reported in Section 3.2.2. Each analysis was performed in triplicate. The cumulative percentage of released proteins and lipids was calculated using the following equation:

Cumulative amount of drug released (%) = C_i_/C_0_ × 100, (1)

where C_i_ is the amount of the proteins/lipids released at a definite time interval, and C_0_ is the loaded protein/lipid amount.

#### 3.3.5. Drug Release Kinetic Study

The in vitro drug release data were interpolated using different kinetic models, as reported below.

Higuchi

*F*(*t*) = *k* × *t*^0.5^, (2)

*F*(*t*) = 100 × (1 − C × exp ^(−*k* × *t*)^), (3)

where *F*(*t*) is the amount of drug dissolved at time *t* and *k* is the release constant. Equation (3) was reproduced from (Equation (2.12) from [32]).

Peppas–Sahlin

*F*(*t*) = *k*_1_ × *t^m^* + *k*_2_ × *t*^(2 × *m*)^, (4)

where *F*(*t*) is the amount of drug dissolved at time *t*, *k*_1_ is the diffusion constant, *k*_2_ is the erosion constant, and *m* is the diffusional exponent, indicative of the drug release mechanism.

Ritger–Peppas

*F*(*t*) = *k* × *t^n^*, (5)

where *F*(*t*) is the amount of drug dissolved at time *t*, *k* is the release constant, and *n* is the release exponent, indicative of the drug release mechanism.

Zero-order

*F*(*t*) = *k* × *t*, (6)

where *F*(*t*) is the amount of drug released in time *t*, and *k* is the release constant.

Korsmeyer–Peppas

*F*(*t*) = *k_KP_* × *t^n^* × Q_0_, (7)

where *F*(*t*) is the amount of drug released at time *t*, *k_KP_* is the release constant, *n* is the release exponent, indicative of the drug release mechanism, and Q_0_ is the initial amount of drug.

### 3.4. In Vitro Evaluation of Lyosecretome Function

#### 3.4.1. Isolation of the Stromal Vascular Fraction (SVF)

SVF was isolated from cryopreserved adipose tissue, previously stored for experimental needs at the skin bank in Turin, Italy. First, 70 mL of lipoaspirates were processed by enzymatic digestion at 37 °C with collagenase NB4 0.3 U/mL using a tube rotator. After 45 min, the enzyme was neutralized by the addition of DMEM low glucose + 10% FBS, and the samples were centrifuged at 1700 rpm for 10 min. The pellet was resuspended in the culture medium, passed through 100 μm and 70 μm cell strainers, washed with PBS, collected, and counted.

#### 3.4.2. Cell Cultures

The SVF cells were seeded on lyosecretome-loaded (SBlyo) and unloaded (SB) SmartBone^®^ samples (*n* = 24 scaffolds for each condition). In detail, 1 × 10^6^ cells were plated on SB in *α*-MEM with 10% *v*/*v* FBS, 2 mM glutamine, and 1% *v*/*v* antibiotics (Gibco, Life Technologies, Milan, Italy) or in osteogenic medium (OM) containing *α*-MEM supplemented with 10% FBS, 50 μg/mL ascorbic acid, 10^−8^ M dexamethasone, and 10 mM beta-glycerophosphate (Sigma-Aldrich, Milan, Italy). The medium was replaced twice a week, and cultures were maintained for 60 days.

#### 3.4.3. Evaluation of the Gene Expression

After 14 and 60 days of cultures, cells were retrieved from SB, and RNA was extracted for gene expression analysis. Briefly, all samples were treated for 45 min at 37 °C with collagenase 0.3 U/mL to destroy the collagen component of connective tissue. Then, a commercial kit (RNeasy Kit, QIAGEN, Milan, Italy) was used to perform RNA extraction. The lysate was passed five times through a blunt 20-gauge needle to make the buffer action more effective. Then, a volume of 70% *v*/*v* ethanol was added to the homogenized lysate and mixed by pipetting. Samples were transferred to an RNeasy spin column, placed in a 2 mL collection tube, and centrifuged for 15 s at 10,000 rpm. The flow-through was discarded. Buffer RW1 was added to the RNeasy spin column, and samples were centrifuged again for 15 s at 10,000 rpm to wash the spin column membrane. The flow-through was discarded. After this, Buffer RPE was added and centrifuged again for 15 s at the same speed. This step was repeated a second time, except centrifugation was done for 2 min to wash the spin column membrane. In the end, RNase-free water was added directly to the spin column membrane and centrifuged for 1 min at 10,000 rpm to elute the RNA. RNA obtained was maintained at 55 °C for 10 min to allow the single helix to stretch and facilitate the measurement performed by spectrophotometer. The amount of RNA was evaluated using a Spectrophotometer (NanoDrop 2000c, Thermo Scientific, Milan, Italy).

One microgram of RNA was converted into single-stranded cDNA by the High-Capacity cDNA Reverse Transcription Kit (Applied Biosystems, Milan, Italy). Quantitative real-time PCR was performed by the CFX96 system (Bio-Rad). The expression of the following genes was tested: osteocalcin (OCN, NM 199173.5), alkaline phosphatase (ALP, NM 000478.5), and collagen 1 (COLL-1, NM 000088.3); the primer sequences were previously published [27,53]. RT-PCR was performed with a SensiFAST™ SYBR Hi-ROX kit (Bioline, Lodi, Italy). The amplification protocol foresees 40 cycles with a T_m_ of 58 °C. The expression of *β*-actin was chosen to normalize gene expression data, and the 2^−ΔΔCt^ method was used for the quantitative analysis with CFX Manager software (Bio-Rad, Hercules, CA, USA).

#### 3.4.4. Micro-CT Analysis

After 60 days, SB and SBlyo were collected, washed with PBS to remove the remaining medium, and fixed in a neutral buffer containing 4% *v*/*v* formaldehyde. Samples were analyzed using high-resolution X-ray microtomography (SkyScan 1172, Bruker, Billerica, MA, USA) to study the structure and quantify the newly formed tissue after SVF colonization [27]. Acquisitions were performed at 80 kV using a 0.5 mm Al filter at a resolution of 6 m, 0.4° of rotation step, 360° scan, and 4× frame averaging. Datasets were reconstructed with NRecon software (Bruker, Billerica, MA, USA). A color contrast mask was used to identify the new formed mineralized tissue. Two expert operators performed quantification on axial slices, measuring the mineralized tissue length using DataViewer.

#### 3.4.5. Histochemical Analyses

After removing all medium traces, SB and SBlyo samples were fixed in a neutral buffer containing 4% *v*/*v* formaldehyde, washed, and decalcified with MicroDec EDTA-based solution by Diapth. Specimens were then dehydrated and paraffin-embedded through EZ Prep Concentrate solution, cut into thin sections, and stained with hematoxylin and eosin (H&E) for morphological analyses. Immunohistochemical analysis was performed using the automated instrument BenchMark ULTRA (Ventana, Milan, Italy). Tissue sections were incubated with the following primary mouse monoclonal antibodies, purchased from Abcam, Cambridge, UK: OCN (ab93876, at 1:250 dilution), transforming growth factor-β (TGF-β) (ab92486, at dilution 1:150), and COLL-1 (ab34710, at 1:400 dilution). Each monoclonal antibody was titrated to yield maximal specific staining and minimal nonspecific (or background) staining. The endogenous peroxidase activity was inhibited by the addition of ultraView Universal DAB Detection Kit. All samples were counter-stained with Mayer’s hematoxylin solution and mounted with Kaiser’s glycerol gelatin. Slides were examined double-blind, and microphotographs were taken using an Olympus BX51 microscope equipped with a digital camera (Nikon DCS E995).

### 3.5. Statistical Analysis

Raw data were processed using STATGRAPHICS XVII (Statpoint Technologies, Inc., Warrenton, VA, USA). A generalized linear analysis of variance model (ANOVA) was used. The differences between the means were estimated by Fisher’s least significant difference (LSD). The statistical significance was set up at *p* < 0.05. Otherwise specified, data are reported as mean values ± standard deviation (from at least three independent experiments).

## 4. Conclusions

This work demonstrated that lyosecretome-loaded SmartBone^®^ (SBlyo) can improve SVF osteoinductive potency. Starting from this result, our strategy has the advantage of using a standardized and ready off-the-shelf MSC-secretome powder, and we obtained, for the first time, a controlled release bioactive bone graft. After plating SVF on SB or SBlyo, tissue growth was monitored at 14 and 60 days, increasing new tissue areas over time. After 14 days, more significant cell proliferation was observed on SBlyo, where cells filled the cavities between the trabeculae. On SB, on the other hand, the process was slower; thus, tissue formation was less organized at 60 days. On both SBs, cells differentiated into osteoblasts expressing ALP and were able to mineralize after 60 days. Nonetheless, SBlyo showed a higher expression of osteoblast markers and a higher quantity of newly formed trabeculae than SB alone. The quantification analysis of the newly formed mineralized tissue demonstrated that SBlyo induces bone formation more effectively. The immunohistochemical analysis performed at 60 days allowed us to observe highly organized bone tissue on SBlyo, synonymous with the fact that cells are stimulated to grow and differentiate by being in an osteogenic environment. This effect presumably depends on the osteogenic factors present in the lyosecretome, such as fibronectin, alpha-2-macroglobulin, apolipoprotein A, and TGF-β.

## Figures and Tables

**Table 1:** Results of in vitro release model fitting for proteins and lipids from SBlyo. Kinetic elaborations were performed on release data obtained from at least three independent experiments for each batch. “~” indicates that the analysis performed was “ambiguous”; therefore, the fit does not nail down the values of all the parameters, and 95% confidence bounds cannot be reported. These latter data were not considered in the interpretation of results.

Model	Equation	Proteins/Lipids	Coefficients (95% Confidence Bounds)	Sum of Squares	*R* ^2^	Degrees of Freedom	SE
Higuchi	*F*(*t*) = *k* × *t*^0.5^	Proteins	*k* = 19.79	3,081,232	0.253	32	1.479
			(16.78, 22.80)				
		Lipids	*k* = 1.498	43,540	−0.2396	32	0.1758
			(1.139, 1.856)				
Higuchi (eq 2.12 from [32])	*F*(*t*) = 100 × (1 − C × exp ^(−*k* ×^ *^t^*^)^)	Proteins	*C* = −4.113	2,103,706	0.49	31	*C*
			(−5.216, −3.066)				0.5425
			*k* = −0.0002381				*k*
			(−0.0003286, −0.0001524)				0.00004424
		Lipids	*C* = 0.5763	28,621	0.1851	31	*C*
			(0.4179, 0.7419)				0.07758
			*k* = 0.0002817				*k*
			(0.0000523, 0.0007763)				0.0001296
Peppas–Sahlin	*F*(*t*) = *k*_1_ × *t^m^* + *k*_2_ × *t*^(2 ×^ *^m^*^)^	Proteins	*k*_1_ = ~				*k*_1_~
			*k*_2_ = ~				*k*_2_ *~*
			*m* = ~				*m~*
		Lipids	*k*_1_ *= ~*				*k*_1_ *~*
			*k*_2_ *= ~*				*k*_2_ *~*
			*m = ~*				*m~*
Ritger–Peppas	*F*(*t*) = *k* × *t^n^*	Proteins	*k* = 248.9	1,208,384	0.707	31	*k*
			(144.2, 394.5)				59.38
			*n* = 0.1725				*n*
			(0.1076, 0.2459)				0.03278
		Lipids	*k* = 33.89	21,454	0.3892	31	*k*
			(14.45, 63.50)				11.55
			*n* = 0.09547				*n*
			(0.001254, 0.2032)				0.04875
Zero-order	*F*(*t*) = *k* × *t*	Proteins	*k* = 0.3309	5,985,916	−0.4512	32	0.0378
			(0.2539, 0.4079)				
		Lipids	*k* = 0.02411	66,142	−0.8831	32	0.003974
			(0.01602, 0.03221)				
Korsmeyer–Peppas	*F*(*t*) = *k_KP_* × *t^n^* × Q_0_	Proteins	*k_KP_* = 248.9	1,208,384	0.707	31	*k_KP_*
			(144.2, 394.5)				59.38
			*n* = 0.1725				*n*
			(0.1076, 0.2459)				0.03278
		Lipids	*k_KP_* = 33.89	21,454	0.3892	31	*k_KP_*
			(15.45, 63.50)				11.55
			*n* = 0.09547				*n*
			(0.001254, 0.2032)				0.04875

## Data Availability

The data presented in this study are contained within the article.

## Supplementary Materials

The following are available online at https://www.mdpi.com/article/10.3390/ijms22084064/s1.
