# Supplementary material for: Biohybrid Bovine Bone Matrix for Controlled Release of Mesenchymal Stem/Stromal Cell Lyosecretome: A Device for Bone Regeneration
Source: Int J Mol Sci. 2021 Apr 14;22(8):4064. doi: 10.3390/ijms22084064 (PMC8071018; doi:10.3390/ijms22084064)
Supplement: Supplementary file 1 [file ijms-22-04064-s001.pdf]

Biohybrid Bovine Bone Matrix for Controlled Release of Mesenchymal Stem/Stromal Cell Lyosecretome: a Device for Bone Regeneration

Elia Bari [1#], Ilaria Roato [2#], Giuseppe Perale [3,4,5], Filippo Rossi [6], Tullio Genova [7], Federico Mussano [2], Riccardo Ferracini [8], Marzio Sorlini [9,10], Maria Luisa Torre [1,10*], Sara Perteghella [1,10]

[1] Department of Drug Sciences, University of Pavia, Viale Taramelli 12, I-27100 Pavia, Italy; elia.bari@unipv.it (E.B.); sara.perteghella@unipv.it (S.P.); marina.torre@unipv.it (M.L.T.)
[2] CIR-Dental School, Department of Surgical Sciences, University of Torino, Via Nizza 230, 10126 Torino Italy; ilaria.roato@unito.it (I.R.); federico.mussano@unito.it (F.M.)
[3] Industrie Biomediche Insubri SA, via Cantonale 67, 6805 Mezzovico-Vira, Switzerland; giuseppe@ibi-sa.com (G.P.)
[4] Faculty of Biomedical Sciences, University of Southern Switzerland (USI), Via G. Buffi 13, 6900 Lugano, Switzerland
[5] Ludwig Boltzmann Institute for Experimental and Clinical Traumatology, Donaueschingenstrasse 13, 1200 Vienna, Austria
[6] Department of Chemistry, Materials and Chemical Engineering "Giulio Natta", Politecnico di Milano, via Mancinelli 7, 20131 Milano, Italy; filippo.rossi@polimi.it
[7] Department of Life Sciences and Systems Biology, University of Torino, Via Accademia Albertina 13, 10123 Torino, Italy; tullio.genova@unito.it
[8] Department of Surgical Sciences and Integrated Diagnostics, University of Genova, Viale Benedetto XV 6, 16132 Genova, Italy; ferracini@edu.unige.it
[9] SUPSI - Department of Innovative Technologies, Lugano University Centre, Campus Est, Via la Santa 1, 6962 Viganello, Switzerland; marzio.sorlini@supsi.ch
[10] PharmaExceed Srl, Piazza Castello 19, I-27100 Pavia, Italy
* Correspondence: marina.torre@unipv.it; Tel.: +390382987779
[#] Equally contributed to the work.

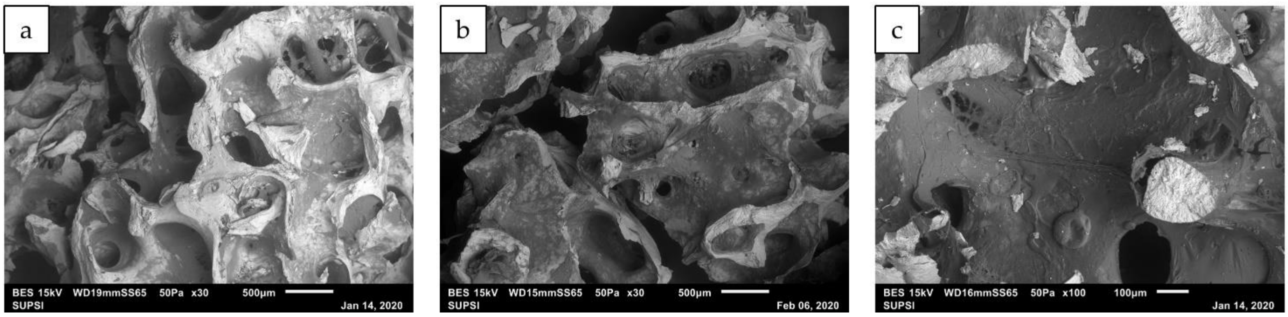

Figure S1. Morphological investigation by ESEM of SmartBone® loaded with 0.1% *w/v* poloxamer 407 (**a-c**). SEM images were taken at increasing magnifications (30× and 100×). Scale bar: 500 and 100 µm.
